# A comprehensive survey of the relationship between self-efficacy and performance for the governmental auditors

**DOI:** 10.1186/s40064-016-2104-x

**Published:** 2016-04-23

**Authors:** Jau-Ming Su, Shue-Ching Lee, Sang-Bing Tsai, Tzu-Li Lu

**Affiliations:** Department of Transportation Technology and Logistics Management, Chung Hua University, No. 707, Sec. 2, WuFu Rd., HsinChu, 300 Taiwan; Ph.D. Program of Technology Management, Chung Hua University, No. 707, Sec. 2, WuFu Rd., HsinChu, 300 Taiwan; Genomic Research Center, Academia Sinica, Taipei, 115 Taiwan; College of Business Administration, Dongguan University of Technology, Guangdong, 523808 China; Zhongshan Institute, University of Electronic Science and Technology of China, Guangdong, 528402 China; Law School, Nankai University, Tianjin, 300071 China; Department of International Trade, Takming University of Science and Technology, Taipei, 114 Taiwan

**Keywords:** Self-efficacy, Professional development, Knowledge sharing, Government auditing, Management

## Abstract

As governmental auditing is involved in evaluating the legitimacy, economy, efficiency, and effectiveness of how the various administrative branches use their allocated resources to optimize the government’s functions, it is expected that the performance of the auditors in charge are strongly influenced by their respective qualities such as self-efficacy and experience, etc. To further understand the factors that may enhance their performance and to ultimately provide practical recommendations for the audit authorities, we have surveyed about 50 % of all the governmental auditors in Taiwan. The result showed that any auditing experience and professionalization do positively influence the professional awareness, and acquired knowledge and skillset of an auditor can effectively improve his or her professional judgment. We also found that perceived ability, problem-solving skills, and resource sharing may significantly impact any performance involved. Our study provides a workable management guidelines for strengthening the self-efficacy of audit authorities in Taiwan.

## Background

The responsibility of government audit authorities is to supervise the implementation of government budgets and evaluate the use of administrative resources to ensure that funding is used wisely, economically, and effectively. A quality auditing system promotes government efficiency. In practice, besides to inspecting existing financial records, government audit authorities should maximize the use of resources by ensuring that the administrative units, departments, and sections of the executive branch achieve their desired goals economically and efficiently. An audit normally concludes with a report to the executive branches with suggestions for improving their work performance. Thus, a quality audit involves reviewing policies according to international standards and perspectives, and provides insight, predictions, and warnings to related organizations. Such practice can reflect the effectiveness of a government.

Under the intense wave of globalization, changes are accompanied by technological innovations, economic liberalization and increasing awareness of an active citizenship, all of which in turn bring about tremendous challenges to the Taiwan government in many frontlines including governmental reform, economic growth, social welfare, tax revenues, anti-corruption, insurance and pension plans. All these issues are strongly influenced by international events, the government’s policies and finance regulations. Since the quality of governmental auditing services not only reflect how the government functions but also influence how the people view their government and its executive branches, auditing performance has become an important approach to increase the value of resources and to stimulate economic development.

There are currently 666 governmental auditors in Taiwan. All of them are either highly educated or richly experienced, or both, as they had first been required to have passed the National Civil Service examinations in accounting and auditing. Most of them are in fact certified public accountants, engineering specialists, or certified internal auditors. The auditors have large workloads. In 2012, they inspected approximately 8800 central and local governmental organizations, and audited the spending of regular and special budgets totaling US$601 billion. On average, each auditor was responsible for 13 organizations and US$902 million. Time constraints and workload pressure can reduce efforts toward knowledge sharing. Workload pressure may degrade both the extent and quality of knowledge sharing among members of an auditing team.

**Fig. 1:**
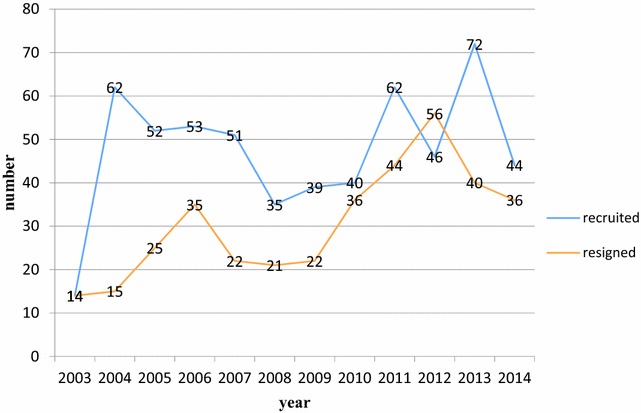
The dynamic changes of personnel between 2003 and 2014

In recent years, the Taiwan government has shifted its auditing focus from a more concern with legality and financial regulation to that of economic, efficient, and effective performance auditing, which is arguably more diverse and complicated. As creating and cultivating a developmental network is an arguably optimal approach to ensure continuous improvement in a rapidly changing work environment (such as globalization, etc.), one such strategy is to develop a mentoring system. High-quality mentoring is characterized by mutual learning, wherein partners can experience an increased sense of work, knowledge, empowerment, and enthusiasm, as well as a desire for more connections. However, due to the stressful working environment, personnel loss has become a key factor causing instability of the structure in these auditing organizations (Fig. 1). Such problem significantly lowers organizational performance. In order to retain auditors, audit authorities must develop an enticing future outlook that emphasizes feedback and learning within the organization.

In professions other than auditors, such as teachers, nurses or social workers, self-efficacy has been used to evaluate job satisfaction, job performance, career development, and health promoting behavior. The findings from these professions demonstrate the importance of self-efficacy for predicting and improving work performance. If an auditing performance is sensitive to ability, effort, and persistence, then efforts made to change self-efficacy (by changing beliefs, information, and knowledge) should improve performance. Although previous researches in auditing accountability, mandate, function, procedures, practices and auditor independence, personality traits, job stress, organizational commitment and intention to leave have been discussed, the relationship between the self-efficacy and auditors’ performance has not yet been fully investigated.

The purpose of this study is to advance the understanding of factors that may enhance auditors’ mission performance and to ultimately provide practical recommendations for the audit authorities concerned. And in order to so evaluate whether mission performance can be improved by the professional development and self-efficacy of auditors, we have conducted a large-scale survey of auditors working for Taiwan government, which have resulted in proposing management recommendations to assist auditors in enhancing professional growth and increasing self-efficacy.

## Literature review

### Self-efficacy and performance

The concept of self-efficacy was developed by Bandura (1977). He proposed that individual behavior is a result achieved from interactions between environment and personal factors. Although self-efficacy is individual’s subjective view of his/her own ability, it profoundly influences personal actions, motivations, persistence, and therefore, the ultimate behaviors (Bandura 1991). Gist and Mitchell (1992) stated that self-efficacy is an important motivational construct. It influences individual choices, goals, emotional reactions, effort, coping, and persistence. Charkhabi et al. (2013) found that when people with high level of self-efficacy are faced with academic problems, they are less likely to give up and would try to find useful solutions to fix the problems. Since self-efficacy is an important construct, it can increase energy, provide direction, and stimulate persistence (Porter et al. 1974). In fact, self-efficacy plays an important role for all professionals, including auditors. Hayati et al. (2014) stated that five job characteristics including skill variety, task identity, task significance, feedback and authority have play a critical role in growing work motivation. For example, Bright (2009) found that public employees such as auditors with high levels of public service motivation were reported to have been significantly more interested in jobs that provided them with personal recognition, task meaningfulness, and professional growth. As auditing is a profession that provides services based on knowledge and experiences with human resource as the key element, the motivation and aptitude of an auditor to accomplish a goal is a strong advantage. As auditors do not work in isolation, it is crucial to understand how the people, tasks, and environment that auditors interact with will influence their auditing performance. Auditors with higher self-efficacy are more likely to continue investing in goal-achievement behaviour. Therefore, self-efficacy will influence behavior by affecting motivation and confidence to overcome difficulties and improve performance.

Vancouver et al. (2001) found a strong correlation between self-efficacy and performance at the personal level of analysis. The strong positive relationship is the result of past performances influence on self-efficacy as described by Bandura (1986). Hoy and Miskel (2001) believed that the past work performance has a significant impact on the individual’s self-efficacy. The continuous success would definitely enhance individuals’ self-efficacy whereas the constant failures would create personal doubt and reduce personal self-efficacy. Since auditors accumulate knowledge and experience from clients to make professional judgments, auditing experience and professionalization can influence professional awareness. Professional judgment determines auditing procedures, and professionalization provides an advantage in client disputes. Nelson (2009) stated that a knowledgeable auditor can identify errors and process complex evidence. Moriarity (1979) found better performance by experienced auditors at bankruptcy prediction, which reflected superior ability of experienced auditors at auditing tasks. Cervone et al. (1991) observed that individuals with high self-efficacy learn more from feedback, respond more adaptively to decision environment, and overtime, are better able to translate their learning into performance. Therefore, to successfully resolve a challenge and complete an auditing task, the problem solver must draw upon experience, knowledge, and cognitive abilities. As specific knowledge is accumulated and more auditing skills are being developed, auditors become more likely to produce professional and comprehensive auditing reports.

Some of the determinants of self-efficacy are well-recognized, attributed causes (i.e., effort, ability, task difficulty). Bandura (1986) pointed out that persistence and level of effort mediated the relationship between self-efficacy and performance. Individuals high in self-efficacy approach difficult tasks as challenges to be mastered rather than as threats to be avoided, set themselves challenging goals, maintain a strong commitment to these goals, and persist in their efforts in the case of a failure. Parker (1998) found that employees who feel capable of performing particular tasks will perform them better, will persist at them in the face of adversity, and will cope more effectively with change. Successful experience not only increases personal expectation on control and maturity of associated actions, but also provides source of self-efficacy for next challenge. Therefore, self-efficacy is widely perceived as one critical factor in determining how much effort and resources a person invests when confronting challenges.

Owhoso and Weickgenannt (2009) found that auditors’ ratings of self-perceived abilities correspond with their actual performance, and these perceptions are influenced by auditing experience and effectiveness when conducting audits within their domain of specialization. Bonner and Lewis (1990) indicated that although more experienced auditors outperform less experienced auditors on average, knowledge and innate ability provide a better explanation of variation in performance. Knowledge is an enduring advantage that is constantly stimulated and accumulated to evaluate new experience and integrate information. From the perspectives both of work specialization and client custom characteristics, an auditor with strong knowledge in the auditing profession is more capable to detect fraud and more likely to allocate resources to recruitment, training, technology, and auditing techniques to improve auditing service quality. One of the strategies to advance a better performance is utilizing the modern technology. Robson et al. (2007) argued that changes in auditing technologies are linked to transformations within the organizational field of auditing. Technological advancements have enabled auditors on engagement teams to conduct electronic reviews of clients’ workpapers in their offices or from remote locations (Brazel et al. 2004). Auditing software reduces the time required for workpapers preparation. Such applications include decision support and expert systems, expert knowledge for specific problems, and point-to-point knowledge. Staff members can access industry best practices, studies, surveys, statistics, expert knowledge for specific problems, and point-to-point knowledge (Silvi 2002). Auditors can work on their personal development through continued learning to accumulate vast knowledge, cultivate open-minded attitude, improve sensitivity, make life plan, and promote peer learning. Overtime, auditors gain more client-specific knowledge, which is positively correlated to audit performance.

Task performance has been defined as work-related to three essential components of any task, namely product of the task, required acts necessary to perform the task, and information cues on which a person can base the judgment about the execution of the task. Stajkovic and Luthans (1998) indicated that self-efficacy is positively and strongly related to work-related performance. The relationship between self-efficacy and work-related performance is moderated by task complexity and locus of performance. Complex auditing tasks typically represent multifaceted construct with different implications for behavioral, information processing, and cognitive facilities of the task performer. Bandura (1986) pointed out that highly complex tasks require different skills necessary for their successful execution by placing greater demands on required knowledge, cognitive ability, memory capacity, behavioral facility, information processing, persistence, and physical effort. Bierstaker and Wright (2001) found that both ability and experience are determinants of performance on an ill-structured analytical review task and an ill-structured internal control auditing task. Ill-structured problems are routinely encountered in auditing. In order to solve an ill-structured problem, a problem solver must draw on experience, knowledge, and cognitive abilities. Therefore, professional development is a learning process that can promote personal growth, improve auditing skills, revolutionize working procedures, and increase auditing report quality.

### Governmental auditors

Auditing is a service profession. Due to many uncertainties of the auditing process and unobservable characteristics of the results, auditing is based on knowledge and experience with human resource as a key element (Knechel 2010). Knechel et al. (2013) stated that audit quality is conceived in different perspectives. For the economic supervision, the audit quality means no major mistakes in the financial report. For auditors, the audit is accomplished according to the methodology or guideline defined by the audit authorities to stand the challenges from the court. The public thinks that audit quality is to prevent a company or a market to cause social economic problems. In general, auditors are exposed to tasks of varying complexity and auditors are assigned to multiple engagements. The higher-complexity task involves a larger number of cues, and more cognitive effort is needed for encoding these cues. The higher-complexity task also involves more processing steps that require cognitive effort in retrieving the requisite knowledge and applying it to the task. The ability of an auditor to perform an auditing task is a major factor in assigning him or her to an auditing team, tasks in order to ensure quality in the conduct of the audit engagement. Thus, auditors need to share their knowledge and expertise about industry-specific trends as well as accounting, auditing, and regulatory issues that may impact the conduct and outcome of the respective auditing team.

Auditors have high knowledge and accountability levels. As task complexity increases, high-accountability audits are likely required to put in the ever more effort to apply the requisite knowledge (which they possess) to successfully perform the task in hand. Tan et al. (2002) suggested that when assigning auditors to highly complex tasks, CPA firms should ensure that the auditors have the requisite knowledge as well as the appropriate motivational level. Auditors assigned to “significant audit responsibilities should possess the knowledge, skill, and ability” to effectively complete those tasks. Specialization has become more important in the current auditing environment, and the auditing team characteristic has evolved into one of the crucial factors for audit quality. Rhee et al. (2013) found that the ability to work effectively on a team is highly valued by employers, and collaboration among employees can lead to intrinsic motivation, increased persistence, and greater transferability of skills. Vera-Muñoz et al. (2006) emphasized the importance of sharing knowledge and expertise among auditing team members to affect a favorable auditing outcome. Nelson and Tan (2005) stated that auditor knowledge and expertise are also associated with superior performance in an auditing, and experience affords opportunities to gain additional knowledge—which when combined with ability—positively affects performance. Consequently, assigning personnel with the appropriate levels of technical training and proficiency to audit engagements is required.

To enhance the quality, effectiveness, and efficiency of the auditing process, the current regulatory environment and new auditing standards in Taiwan have broadened and intensified pressures on auditing governmental authorities and other non-official agencies. Because of such stressful working environment, personnel loss is a key factor which cause instability of the structure in the audit organizations (Fig. 1). Such problem significantly lower the organizational performance. Many studies have demonstrated that level of self-efficacy can predict work attitudes, job training, work performance, job satisfaction, educational development, and knowledge sharing (Randhawa 2004; Hsu et al. 2007; Hoy and Miskel 2001; Cabrera et al. 2006). The National Audit Office of the Taiwan aims to evaluate the audit quality in the following five areas: leadership, personnel, auditing, clients, and continual improvement. Based on these areas auditors are expected to provide independent insight and to be forward-looking, as well as offering advice to improve the efficiency of government authorities they serve.

## Methods

### Survey participants

As of December 2014, there were 666 auditors in Taiwan government. All auditors surveyed in this study are highly educated, experienced, or both, and required to pass the National Civil Service Examinations in accounting and auditing. Most of their job titles are auditors or senior auditors.

The participants surveyed in this study all are employees of either the Central or local government audit authorities, including the National Audit Office and its subsidiary Audit Divisions and Offices, the Education and Agriculture Audit Division, the Construction of Transportation and Communication Audit Division, the Five Municipality Audit Divisions, and the 16 county Audit Offices. The survey was conducted using the stratified random sampling method from April to June 2013.

### Questionnaire design

The survey questionnaire comprised two parts. The first part included the collecting of basic personal information, such as gender, age, education, years of auditing, division affiliation, and job title. The second part included questions about self-efficacy, organization culture, intrinsic motivation, and mission performance, and was designed on the basis of principal component factors and varimax rotation factor analysis so as to ensure that the questions can cover every conceivable perspective.

All questionnaire items were measured using a 5-point Likert-type scale (1 = *strongly disagree* to 5 = *strongly agree*). Nine items were designed according to the self-efficacy scales designed by Schwarzer and Jerusalem (1995). Eight items were based on factors related to organization culture and intrinsic motivation, including learning motivation, as addressed by Ames (1992); knowledge sharing, as addressed by Hendriks (1999); and organization culture, as addressed by Wallach (1983). Fourteen items were based on the auditing-related mission performance; thus, the reliability of each structure was ensured (Nunnally 1978, Bagozzi and Yi 1988). All of these attributes were developed from information in the literature review and personal interviews with auditing-related assistant auditor generals and senior auditors. A pilot test was conducted by interviewing 6 senior auditors from New Taipei Municipality Audit Divisions to assess the reliability of the self-efficacy and auditing performance attributes. Some wordings in the questionnaires were rephrased to clarify the questions after the pilot test. And the items listed on the final survey were examined carefully to avoid repeated questions. Expert validity and Cronbach’s alpha coefficients were used to examine reliability and validity of the questionnaire. Cronbach’s alpha coefficients for self-efficacy, organization culture, and mission performance were 0.88, 0.85, and 0.92 respectively (Table 1).

**Table 1:** The reliability of each structure

Variable name	Factor name	Factor loading	Variance explained	Cronbach *α*	Overall Cronbach *α*
Self-efficacy	Problem-solving skills	0.587–0.786	52.486	0.810	0.884
	Perceived ability	0.617–0.852	9.382	0.761	
	Confidence and persistence	0.655–0.877	8.927	0.734	
Organization culture	Learning culture	0.839–0.894	39.684	0.846	0.846
	Resource sharing	0.542–0.872	34.401	0.733	
Mission performance	Mission outcome	0.649–0.793	48.419	0.882	0.915
	Teamwork	0.688–0.841	10.512	0.825	
	Job objectives	0.755–0.803	6.843	0.845	

In the questionnaire, self-efficacy consists of three dimensions: problem-solving skills, perceived ability, as well as confidence and persistence. Intrinsic motivation acquisition has three dimensions: development growth, accomplishment, and knowledge learning. Organization culture consists of two dimensions: learning culture and resource sharing. Mission performance comprises three dimensions: mission outcome, teamwork, and job objectives.

### Sampling accuracy evaluation

The audit authority of the Taiwan comprises 36 assistant auditor generals (5.4 %), 277 senior auditors (41.6 %), 77 senior inspectors (11.6 %), 231 auditors (34.7 %), and 45 inspectors (6.7 %). Because the number and titles of government auditors’ positions are regulated by the law concerned, the accuracy of sampling can be evaluated based on the job titles of the respondents to ensure that our sample represented a subpopulation of the overall audit authority. A Chi squared test with *p* > .05 indicates no significant difference between our sample structure and the matrix structure.

### Structural equation model analysis

**Fig. 2:**
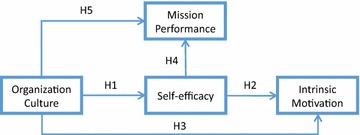
Research framework

The structural equation modeling (SEM), a multivariate statistical technique for testing structural theory, was used to test the proposed model and hypotheses. This approach incorporates both observed and latent variables. The analysis for this study was conducted using LISREL 8.50 and by utilizing the maximum likelihood method. In the proposed model (Fig. 2), organization culture was considered to be an exogenous variable, while mission performance and intrinsic motivation were regarded as endogenous variables, and self-efficacy was taken both as an endogenous and exogenous variables.

The research framework of this study is illustrated in Fig. 2; the arrows indicate the direction of influence among the components. Among these variables, self-efficacy is widely thought to have a significantly positive correlation with performance in different fields. In this study, we investigated whether such relationship exist between auditors and mission performance. After surveyed government audit staffs at various levels in Taiwan, we then used a survey research method to examine the hypothesized relationships among organization culture, self-efficacy, intrinsic motivation, and mission performance.

## Results

### Responses

Of the 339 questionnaires distributed to active auditors in Taiwan, 326 valid responses were returned, representing a very high response rate of 96.17 % and a sampling ratio of 48.95 %.

### Fair sample structure–matrix structure alignment

Our sample comprised 10 assistant auditor generals (3.1 %), 146 senior auditors (44.8 %), 31 senior inspectors (9.5 %), 118 auditors (36.2 %), and 21 inspectors (6.4 %). The survey results were not varied significantly according to job title (*p* > .05, Chi squared test), indicating that the survey sample structure is aligned with the matrix structure.

### Current state of each factor

The results of the response averages for all dimensions revealed that “knowledge learning” obtained the highest score (3.94), whereas “confidence and persistence” obtained the lowest score (3.29). Among the nine statements in the “self-efficacy” section of the survey, “I can always solve a problem no matter how hard it is” received the most agreement (3.86); “I am confident in handling my work” (3.48) and “I can easily stick to and achieve goals” (3.10) obtained lower score. In the “organization culture” section, “My organization has established an internal network to share knowledge and experience” received the most agreement (3.76); “My organization has adequate knowledge resources” (3.31) obtained lower score. In the “intrinsic motivation” section, “My work provides opportunities to learn new knowledge” (3.94) received the most agreement. Among the 14 statements in the “mission performance” section, “I am happy to assist colleagues in solving work problems” has received the most agreement (3.88); “I have good oral communication skills” (3.46) and “I am sensitive to the development of new technology and know how to apply it in my work” (3.36) obtained lower score.

### Demographic variable adjustments

The survey subjects in this study can be categorized on the basis of the following factors (Table 2): (1) gender [women (52.1 %), men (47.9)]; (2) age [under 30 years (6.4 %), 31–40 years (40.5 %), 41–50 years (38.7 %), 51–60 years (10.8 %), 60 years above (3.6 %)]; (3) education level [master’s degree (51.2 %), bachelor’s degree (44.0 %)]; (4) auditing experience [more than 15 years (33.4 %), 11–15 years (17.0 %), 5–10 years (20.1 %), less than 5 years (29.5 %)], and (5) division affiliation [in local city or county Audit Offices (46.0 %), city Audit Divisions (30.1 %), National Audit Office (16.9 %), Audit Division on Education and Agriculture (7.0 %)].

**Table 2:** Sample demographics of the poll (n = 326)

Item	Percentage
Sex	
Male	47.9
Female	52.1
Age	
Under 30	6.4
31–40	40.5
41–50	38.7
51–60	10.8
60 above	3.6
Job title	
AAG	3.1
SA	44.8
SI	9.5
A	36.2
I	6.4
Auditing experience	
Under 5 years	29.5
5–10	20.1
11–15	17.0
15 above	33.4
Education	
Prof. School	4.8
Bachelor	44.0
Master	51.2
Division	
NAO	16.9
EA/TC	7.0
5 CAD	30.1
16 CAO	46.0

*AAG* assistant auditor general, *SA* senior auditor, *SI* senior inspector, *A* auditor, *I* inspector, *NAO* National Audit Office, *EA/TC* Audit Division on Education and Agriculture, Audit Division on Construction of Transportation and Communication, *5CAD* 5 City Audit Division, *16 CAO* 16 County Audit Offices

We performed a differentiated analysis of the individual background variables of the respondents regarding their perception of self-efficacy, organization culture, intrinsic motivation, and mission performance. The results (Table 3) showed that gender is a significant factor in *perceived ability*, *confidence and persistence*, and *mission outcome* (t = 2.73, 4.08, and 3.78, respectively).

**Table 3:** The differentiated analysis of gender, education, division, age, experience, and title

	Gender (*t* value)	Education (*F* value)	Division (*F* value)	Age (*F* value)	Auditing experience (*F* value)	Job title (*F* value)
Problem-solving skills	1.51	2.45	1.53	6.27***	9.25***	10.99***
Perceived ability	2.73**	4.46*	2.54	15.11***	25.73***	16.70***
Confidence and persistence	4.08***	3.55*	1.06	4.23**	5.97***	6.53***
Learning culture	2.27*	0.06	2.58	3.89**	2.96*	6.01***
Resource sharing	1.77	0.76	1.54	3.57**	4.28**	5.20***
Intrinsic motivation	1.68	2.46	1.77	0.55	1.17	4.58**
Mission outcome	3.78***	1.67	0.48	16.68***	24.92***	19.48***
Teamwork	2.18*	1.35	2.67*	8.78***	11.61***	10.87***
Job objectives	1.96	1.34	0.77	9.18***	10.98***	8.40***

* *p* < .05; ** *p* < .01; *** *p* < .001

Because of the background requirements and conservative organization culture of the auditing profession, promotion and job performance are associated with seniority. Thus, the age of respondents was found to be highly correlated to seniority and job titles (r = 0.736, 0.624, and 0.749, respectively). Therefore, we performed an analysis of variance on various age groups on the basis of different years of experience and job titles (Table 3). The results of a post hoc analysis of age demonstrated that older auditors have advantages in *perceived ability* (the auditors in the age group of 30 years old or younger have presented significantly lower results than those who are older). Moreover, auditors who have more years of experience have higher *perceived ability*, *confidence and persistence*, *teamwork*, *mission outcome*, and *job objectives*. (The group with more than 15 years of auditing experience presented significantly higher results than those who were less experienced. As expected, the group with less than 5 years of experience presented the lowest results.)

Regarding job titles, assistant auditor generals, who are competent and have vast practical experience, have exhibited more advantages than auditors and inspectors who in turn scored higher than assistant auditors and assistant inspectors did in *problem*-*solving skills*, *perceived ability*, *confidence and persistence*, *learning culture*, *resource sharing*, *intrinsic motivation*, *mission outcome*, *teamwork*, and *job objectives*.

### Mission performance impact analysis

The results of stepwise regression analysis of self-efficacy (problem-solving skills, perceived ability, as well as confidence and persistence), intrinsic motivation (development growth, achievement, and knowledge learning) and organization culture (learning culture and resource sharing) on mission performance indicated that (a) perceived ability, (b) problem-solving skills, (c) resource sharing, and (d) confidence and persistence significantly positively affected mission performance. The obtained F-ratio for the significance of multiple R was equal to 65.08. The square of multiple R (R^2^) was .61 suggesting that all the eight predictors jointly accounted for 61 per cent of the total variance in mission performance. Among these factors, perceived ability, problem-solving skills, and resource sharing were the most crucial ones. Because of the rapid development of science and technology, performance audits are now being associated with numerous fields, such as the policy sciences. A competent auditor is an expert on audit theory and has expertise in combining technological, managerial, and other relevant information on technology concepts to design a more efficient work process, thereby reducing workloads and allowing more time for training.

The detail analysis of each aspect in self-efficacy indicated that *problem*-*solving skills*, *perceived ability*, as well as *confidence and persistence* have a significantly positive impact on *mission performance*. Therefore, self-efficacy has a significantly positive impact on mission performance (*β* = 0.635, *p* < .001, explanatory power = 57 %).

### Structural equation model analysis

Structural equation modeling was used to test the proposed model and hypotheses. LISREL model revealed a satisfactory fit for our sample data (Table 4).

**Table 4:** Reliability measures

Construct	Observed variable	Standardized factor loadings	Residual	CR	AVE
Self-efficacy	Problem-solving skills	0.81	0.35	0.84	0.64
	Perceived ability	0.82	0.33		
	Confidence and persistence	0.76	0.42		
Organization culture	Learning culture	0.79	0.37	0.81	0.68
	Resource sharing	0.86	0.26		
Intrinsic motivation	Knowledge learning	0.75	0.44	0.88	0.71
	Development growth	0.88	0.23		
	Achievement	0.89	0.21		
Mission performance	Mission outcome	0.84	0.29	0.81	0.59
	Teamwork	0.71	0.50		
	Job objectives	0.75	0.43		

*CR* composite reliability, *AVE* average variance extracted

**Fig. 3:**
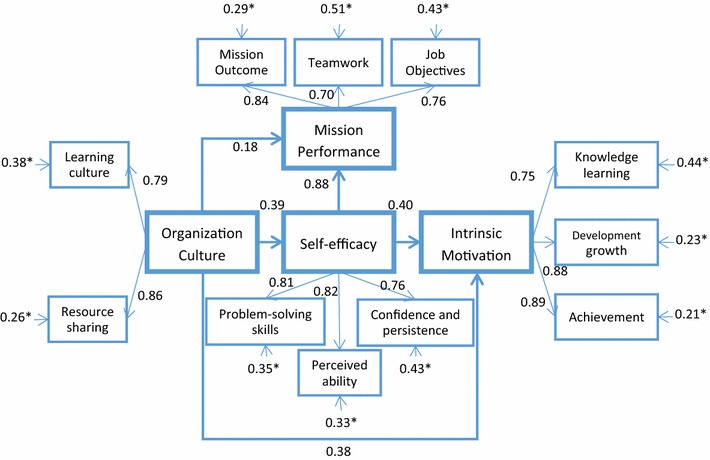
Results of theoretical model analysis

Figure 3 presents the final SEM model analysis. The absolute fit measures (GFI = 0.95, AGFI = 0.91, RMR = 0.047) indicated that the structural model met the recommended levels and thus fitted the sample data collected satisfactorily. The Chi squared statistic divided by the degrees of freedom yielded a reasonable fit of 2.61. Thus we concluded that the proposed model maintained good construct validity. The statistics of the fit test of the model is described in Table 5.

**Table 5:** Fit test of the model

Indicators	Measures
Normed chi-square 101.95/39 = 2.61	<3
Goodness of fit index (GFI) = 0.95	>0.9
Adjusted goodness of fit index (AGFI) = 0.91	>0.9
Root mean square error of approximation (RMSEA) = 0.070	<0.05
Root Mean Square Residual (RMR) = 0.047	<0.05
Normed fit index (NFI) = 0.95	>0.9
Non-normed fit index (NNFI) = 0.96	>0.9
Comparative fit index (CFI) = 0.97	>0.9

## Discussion

Audits optimize government function by evaluating the legitimacy, economy, efficiency, and effectiveness of how administrative branches utilize resources. For example, audit authorities have revealed that the Taiwan government previously constructed so-called “mosquito buildings” (idle buildings “used only by mosquitoes”) to create an illusion of public construction achievement and opportunities for kickbacks. Such construction squandered and unevenly distributed government funds. The number of mosquito buildings reached of 163, but an effective auditing has reduced this figure to nine. Auditing reports can effectively provide opinion and suggestions to further improve the performance of the executive branches. The key to quality auditing is to review policies from an international perspective so as to provide insight, predictions, and warnings for comparison. The purpose of this study was to examine the relationships between self-efficacy, intrinsic motivation, organization culture, and mission performance. The ordinary least squares (OLS) to perform multiple regression analysis has been utilized to calculate the coefficient estimates. Self-efficacy (*β* = 0.327, *p* < .001) is found to have been influenced by Organization culture, and thus supporting H1 as described in (Fig. 2). As predicted in H2, self-efficacy has influenced intrinsic motivation (*β* = 0.473, *p* < .001). The self-efficacy of employees is adjusted on the basis of intrinsic motivation and their organization culture. Intrinsic motivation and mission performance have been found to have been influenced by organization culture (*β* = 0.448, 0.398, *p* < .001), and thus supporting H3 and H5. Finally, mission performance has been found to have been influenced by self-efficacy (*β* = 0.756, *p* < .001), and thus supporting H4. Self-efficacy and mission performance have found to have a positive correlation. High self-efficacy typically leads to higher performance, and self-efficacy has immense effects on an individual’s motivation, effort, persistence and performance. Therefore, self-efficacy is one big key factor in achieving success.

We have found that auditors generally have a positive attitude toward their professional abilities and experience, confirming the view that experience can increase self-efficacy. Self-efficacy obviously depends on past successful experiences, also known as mastery experiences (Bandura 1986). More importantly, achievements foster self-efficacy only when individuals ascribe these successes to their own personal effort or ability (Tolli and Schmidt 2008). In the “intrinsic motivation” section of the survey, “My work provides opportunities to learn new knowledge” (3.94) has exhibited the most agreement. The results have revealed that most of the respondents have believed that numerous opportunities are available for personal growth within their organizations, probably because of the diverse jobs available in auditing. This is because auditors are routinely assigned tasks that vary in complexity and industry. Job enrichment provides a sense of control over one’s work environment and motivates people to exercise their full potential, thus presenting more opportunities for employee success. However, in the “organization culture” section, “My organization has adequate knowledge resources” (3.31) has lower score. The respondents have perceived that audit authorities have not provided adequate knowledge resources. Audit authorities must create, integrate, share, and use knowledge regarding their clients’ control activities and corporate governance. Implementing these knowledge-based activities effectively is increasingly critical for audit authorities in maintaining their competitive advantages, including gaining tangible benefits regarding time and cost reductions. Thus, sharing knowledge can aid audit authorities in leveraging the skills, knowledge, and optimal practices of their professional staff members. Auditors must share knowledge and expertise in industry-specific trends with members of the audit team as well as their accounting, auditing, and regulatory concerns that may influence the performance and outcome of an audit.

In the specific case of audit engagements, members of an audit team typically perform only a discrete part of the audit process. Thus, the knowledge and expertise in the client environment, industry, and interactions with the client’s operations are unevenly distributed among members of the audit team. In the “mission performance” section, “I am sensitive to the development of new technology and know how to apply it in my work” (3.36) has lower score. Auditors have been found to be less confident in remaining abreast of technological developments, thus indicating that audit methodologies, work procedures, and communication with clients could be improved further. Working in a diversified environment, auditors must be administrative and management experts as well as internal-control designers for (a) adhering to audit principles and learning advanced auditing methods, (b) nurturing a sense of innovation for developing novel audit processes and methods, and (c) creating new audit-operating mechanisms. Appropriate use of IT assets can result in organizational innovations and facilitate redesigning business processes, while favorable competitive dynamics can generate improvements in organizational performance. Efficient and effective auditors are required to have continual education and training in mentoring and knowledge sharing. An audit quality reflects a carefully designed audit process that involves recruiting talented auditors to be properly motivated and trained, thus enabling them to understand inherent uncertainties and adjust audit strategies to accommodate unique client conditions. Therefore, increasing the value of the audit profession in a complex audit environment necessitates constantly adapting to new types of technology and updating auditing concepts.

We have discovered that the experience effect is significant in auditing. Their abundant practical auditing experience and excellent performance history have enabled them to understand the profession and culture more in depth, and to advance professional development programs that inspire learning, stimulate knowledge sharing, and promote self-realization. Auditing experience and professionalization have been found to have positive influence on professional awareness, which, in addition to an auditor’s knowledge and skillset, can no doubt improve professional judgment.

The results of a post hoc analysis on age revealed that older auditors have advantages in *perceived ability* (the auditors in the age group 30 years or younger) presented significantly lower results than others. Moreover, auditors who have more years of experience have higher *perceived ability*, *confidence and persistence*, *mission outcome*, and *job objectives*. Shrivastava and Purang (2011) indicate that feedback is effective in the presence of a strong link between performance improvement and valued outcomes. Experienced baby boomers are rapidly nearing retirement age, and their accumulated wisdom and expertise could soon be inaccessible. Brown and Duguid (2000) found that the loss of professional autonomy associated with structured audit approaches has increased the turnover rate among senior audit staff members and, by extension, resulted in the loss of knowledge possessed by exiting personnel. Leaders should create an open culture that is conducive to mentoring, where people learn from one another through a wide variety of formal and informal relationships at an enterprise level. Thus, everyone can reap the benefits of mentoring. Specifically, mentoring others or sharing knowledge can improve the efficiency and effectiveness of audit procedures.

In the “self-efficacy” section of the survey, “I am confident in handling my work” (3.48) and “I can easily stick to and achieve goals” (3.10) were scored lower. Accordingly, the proposed methods for increasing the self-efficacy of auditors has included the following: (a) successful experience from past assignments; (b) self-confidence in one’s potential for achieving goals; and (c) understanding that a performance audit is not a difficult task, but a favorable success model. Therefore, audit tasks should be rotated among staff members for enabling effective cross-training. In this manner, staff members would become more versatile and strengthened by learning from one another, and auditors’ self-efficacy could thus be increased for maintaining high audit sensitivity. Assessments that are more insightful could be performed to recommend alternatives that are more efficient and economical for executive branches to further increase government performance.

Because of the rapid development of science and technology, better performance audits are now being associated with numerous fields, such as the policy sciences. *Perceived ability* arguably exerts the most significantly positive impact on audit performance, followed by *problem*-*solving skills*, *resource sharing*, and *confidence and persistence*. Thus, training multidisciplinary auditors is considered as an urgent task. A competent auditor is an expert on audit theory and has expertise in combining technological, managerial, and other relevant information on technology concepts to design a more efficient work process, thereby reducing workloads and allowing more time for training.

The auditing process usually concludes with a report, which is a compilation of reports from each member of the field task unit. Thus, teamwork affects the quality of audit reporting and requires team leaders to guide every member. Leaders should recognize that developing cooperative relationships among team members can promote team effectiveness. For example, group responsibility for completing challenging tasks, as well as team recognition, can help members’ commitment to do cooperative goals (Chen and Tjosvold 2014). By developing a strong, organized environment, every individual would devote more effort to their work, leading to higher self-efficacy. Therefore, teamwork has been proven to be a significant factor affecting the quality of an audit report. In contrast, an effective audit recommendation should be based on evidence that practically resolves issues in accordance with regulations. When they lack on-the-job training, auditors are incapable of issuing a fair judgment and thus fail to deliver a report, which would adversely reflect on how to effectively use a budget. Performance management can be improved by developing visionary thinking and providing constructive recommendations in an audit report. An audit report can provide information and useful references for further improving the performance of executive branches, thus preventing redundant “mosquito” museums, harbors, and facilities from being built.

To improve communication among audited units, audit authorities should understand that reprimanding people or challenging policies is not the main purpose of a performance audit. Auditors should focus on the outcomes of a policy to obtain insightful results and recommendations for governing without interfering with the executive branches. Therefore, audit authorities can positively affect society by increasing the economic value of audited units. Because the main aim of a performance audit is to improve government performance, audit authorities should strive to develop collaborative partnerships with the executive and legislative branches in enhancing governance. Audit leaders should recognize that developing cooperative goals among team members is essential for reinforcing these values and ensuring the credibility. Our study has showed that the evaluation, performance, and value of outputs and outcomes have evidently relied on auditors’ self-efficacy. Auditors should maintain a strong and professional relationship with audited units, enabling them to appropriately communicate audit results, thereby effectively improving governing performance. According to the mission statement of the American Accounting Association, the auditing profession should “foster excellence in the teaching, research, and practice of auditing and assurance services.” Accordingly, the core competencies of auditors are as follows: (a) communication ability and leadership for inspiring people to achieve common goals; (b) the ability to conduct comprehensive financial analyses, provide insight, and offer constructive recommendations; and (c) an awareness of new technological trends and the ability to apply advanced technology to increase client and employee value. Audit authorities should operate on the basis of public governance perspectives and focus on regulatory and performance auditing to ensure that the executive branches utilizes the allocated funds legally, enabling it to economically and efficiently achieve the set goals.

## Conclusion

After having surveyed nearly 50 % of all the governmental auditors in Taiwan, we have found that experience to be one most significant factor in their auditing efficacy. Auditing experience and professionalization have positively influenced professional awareness, which, with the knowledge and specialty of an auditor, can improve professional judgment. However, senior auditors have been observed to have responded to workload pressure by expending little or no time in providing feedback to audit staff members under their charge. Also, auditor turnover has reduced knowledge sharing in an audit. On the basis of these results, we recommend that the Taiwan government improve its current working environment and enhance job training. To foster knowledge sharing, recruitment and selection should favor people who are open to learning and using novel concepts and practices. Audit authorities should include specific guidance in their recruiting policies that will aid recruiters in identifying candidates who exhibit individual-level traits consistent with the organization’s goal and values that are commonly associated with the ability to work effectively in teams and share knowledge. Therefore, to retain auditors for governmental service, audit authorities must develop an attractive future outlook that emphasizes feedback and learning within the organization.

Audit authorities benefit from the implicit knowledge of their employees; people are the most crucial information carriers and the most abundant assets in governmental audit authorities. Most respondents have believed that there are numerous opportunities for personal growth in their organizations and that their jobs have provided opportunities for future development. However, they have also believed that audit authorities have not provided enough knowledge to enable them to share auditing experiences and educational training. Organizations can begin using technology to help people forge new relationships across traditional boundaries in order to expand learning networks. Making use of peer coaching, mentoring circles, and learning partners can provide favorable opportunities for individuals to build their own developmental networks. To enhance knowledge sharing between preparers and reviewers in the work-paper review process, training should be tailored to the specific needs of different ranks of auditors. Audit authorities should encourage knowledge sharing, strategic job rotation, and cross-training among different generations to integrate baby boomers’ experience with the creativity of Millennials. Such practice can result in groundbreaking innovations.

Overall, the survey findings have indicated that self-efficacy has significantly influenced mission performance. Employers should focus on improving employee self-efficacy for enhancing both individual and organizational performance. When they have assisted each other in achieving their tasks and goals, they would have felt the needed individual support being fully vindicated. Employee self-efficacy can be vicariously enhanced through counseling, job enrichment, proper guidance training, development programs, challenges, and autonomous jobs and rewards. Regarding the results with lower score in our survey, in addition to establishing an internal network, organizations should hold frequent formal meetings to discuss and share knowledge and experience. They should also provide a knowledge-sharing platform and educational training to improve efficiency, thereby increasing the available training to enhance audit quality. When employees believe that they are advancing, their work is meaningful, and their working environment is conducive to develop, their teams are well-structured, and they have sufficient resources, they are most likely motivated to receive more training for better development and efficient execution. Supporting the professional growth of staff members will definitely improve their task performance and personal satisfaction.

To avoid discouraging auditors from responding to our survey, we had not included several performance variables such as recommendations and balances in audit reports, amounts returned to the national treasury, and the number of staff members who had been reprimanded. And future studies can include these variables for comparison. Arguably, auditing performance is difficult to define and quantify, and we recommend future research using alternative measures of mission performance to validate and elaborate on our findings. Several factors examined such as organization culture, self-efficacy and intrinsic motivation in this study are likely to affect mission performance directly, whereas others are more likely to mediate or moderate mission performance. We will encourage future research that empirically examine direct, moderating, or mediating effects on mission performance so as to obtain a more comprehensive picture of good audit quality with respect to the area covered in this study.
